# Effect of Antioxidant Therapy on Oxidative Stress in Vivo

**DOI:** 10.3390/antiox10030344

**Published:** 2021-02-25

**Authors:** Anna Maria Fratta Pasini, Luciano Cominacini

**Affiliations:** Department of Medicine, Section of General Medicine and Atherothrombotic and Degenerative Diseases, University of Verona (Italy) Policlinico G.B. Rossi, Piazzale L.A. Scuro 10, 37134 Verona, Italy; luciano.cominacini@univr.it

Over the last few decades, many efforts have been put into fields that explore the potential benefits of antioxidants, especially with regards to aging, cancer, cardiovascular diseases, and neurodegenerative diseases [1,2,3,4]. However, despite the positive results in experimental studies, there is no clear evidence of clinical benefit of antioxidant supplementation. As reviewed by Vassalle et al. [5], the lack of positive results are likely due to factors related to bioavailability and pharmacokinetics of antioxidants, as well as trial design and patient selection. In fact, antioxidants are likely to be more effective in patients with early damages and in those with increased oxidative stress (OS) status [5,6,7]. On the other hand, OS status assessments are challenging because reliable OS biomarkers still lack [5].

Considering the key physio-pathological role of OS in many chronic diseases and in cancer (notwithstanding the results of clinical trials), there is still a need to continue in the field of OS research and antioxidant supplementation in order to tailor medical management to individual patient characteristics. This Special Issue concerning "The Effect of Antioxidant Therapy on Oxidative Stress in Vivo" includes 13 contributions, 9 research articles, and 4 reviews.

Since ultraviolet (UV) radiation is a well-known risk factor for OS-induced skin injury [8], it has been suggested that antioxidant therapy may be an important tool for counteracting the harmful effects of UV. In this issue, Łuczaj et al. [9] explored whether topical cannabidiol (CBD), a phytocannabinoid with antioxidant and anti-inflammatory effects, can prevent keratinocyte phospholipid profile alteration (using liquid chromatography-mass spectrometry analysis) in nude rat skin exposed to UV radiation. These first in vivo results are very attractive since CBD has been proven to prevent phospholipid oxidation and improve transepidermal barrier status, preventing excessive water loss typical of psoriasis and other skin diseases [10].

Senescence and mitochondrial dysfunction are the main causes for human corneal endothelial cells (CECs) diseases in which corneal grafting is required to treat the condition [11]. Sirtuin 1 (SIRT1) delays aging by lowering mitochondrial OS and regulating mitochondrial respiratory chain [12]. The results of another article in this issue show that senescence is associated with SIRT1 reduction in CECs and in an animal model [13]. Interestingly, SIRT1 activation using the CRISPR/dCas9 gene therapy suppressed cytokine-induced cell death and senescence, thus promoting CEC regeneration [13]. The conclusion is that SIRT1 activation therapy may serve as a novel treatment strategy for CEC diseases.

Sciskalska et al. [14] assessed the impact of acute pancreatitis (AP) on changes in the concentrations and activities of all three superoxide dismutase (SOD) isoenzymes and the interrelationships between the different isoenzymes and the oxidative and inflammatory status. Finally, the effect of single-nucleotide polymorphism (SNP) in the SOD1 gene (rs2070424) was also taken into consideration. The results show that in OS conditions induced by inflammation, the participation of individual forms of plasma SOD isoenzymes in the total antioxidative activity of SOD changes. SNP rs2070424 in the SOD1 gene impacted on total activity of SOD in AP patients which was accompanied by increased inflammatory status. 

Abbas et al. [15] aimed to evaluate the phytotherapeutic ability of propolis (PR) to alleviate the oxidative stress and immunosuppression imposed by an avian pathogenic *Escherichia (E.) coli* using laying hen as a base model. Recently, there has been an increasing interest in replacing antibiotics with alternative natural products [16], such as PR, a resin product of honeybees with rich bioactive antioxidant and bactericidal compounds [17]. The results showed that E. coli challenge worsened antioxidant and immune status, as well as productive performance in hens. These negative effects were fully counteracted by PR administration, suggesting that PR can effectively be used as an organic feed additive to overcome the endogenous OS induced by endotoxin challenge.

By using oxazolone, a hapten experimentally used to induce atopic dermatitis (AD) [18], Yoo et al. [19] examined the impact of cardamonin (C) a naturally-occurring compound with anti-inflammatory, vasodilatative, and anti-infectious actions [20] on oxazolone-induced AD in mice. The results show that topical application of C onto the ear of mice inhibited oxazolone-induced AD in vivo by suppressing the generation of T helper 2 cytokines and the following oxidative injuries through the activation of NRF2, so indicating that C may be beneficial when treating AD.

In a double-blind crossover trial, Capó et al. [21] evaluated the effects of dietary nitrate on the circulating concentrations of oxylipins and cytokines in response to acute moderate exercise in metabolic syndrome patients. Nitrate enriched beverages increased the concentration of nitrite and nitrate in the mouth and plasma, counteracting the negative effects of acute exercise on inflammatory response and oxylipins. These data indicate that ingestion of nitrate-rich foods or nitrate-enriched beverages before practicing exercise may help prevent the inflammatory response associated to exercise in patients with metabolic syndrome.

Zelber-Sagi et al. [22] aimed to examine the relationship between malondialdehyde (MDA), as a marker of lipid peroxidation and non-alcoholic fatty liver disease (NAFLD), as well as liver damage markers of steatohepatitis (NASH) and fibrosis. In addition, they sought to assess the association between dietary vitamins E and C intake, as well as MDA levels in a cross sectional study among healthy subjects. Serum MDA was strictly related with NAFLD and markers of NASH or fibrosis among men. Dietary vitamin E intake appeared to prevent high MDA levels among women, indicating that it may play a role in the protection from NAFLD and primarily NASH in women.

Peritoneal dialysis fluids may have cytotoxic effects on human peritoneal mesothelial cells (HPMCs). Based on their high glucose and lactate concentrations, the acidic pH and numerous toxic glucose degradation products they generate the so-called Amadori adducts [23]. In this scenario, Sánchez-Rodríguez et al. [24] examined the potential cytoprotective effect of polyphenols on HPMCs isolated from patients and exposed to Amadori adducts. It was shown that polyphenols, through their anti-inflammatory and antioxidative impact, may represent a therapeutic tool to reduce complications associated with peritoneal dialysis, thus helping to maintain peritoneal membrane function longer.

Starting from the point that antioxidant dietary intervention may be a potential strategy in postponing age-related diseases, Rusu et al. [25] analyzed the antioxidant actions of walnut kernels (WK) and walnut septum extract (WSE) in a D-galactose (D-gal)-induced aging and in naturally aged rat models. Dietary supplementation with WK or WSE, through their counteracting effects on OS, drove back the typical liver and brain modifications related to aging, observed in a D-gal aging model, as well as in naturally aged animals. 

The review by Berretta et al. [26] considers the pharmacological profile of ascorbic acid (ASC) and its effects on cancer survival, as well as cardiovascular and infectious diseases. Although experimental studies indicate that ASC supplementation may reduce OS and prevent several chronic conditions, the lack of accurate clinical trials prevents drawing clear conclusions on its therapeutic role in critically ill patients. A likely explanation of these negative results may be the scarce understanding of its numerous roles that has produced design imperfections, misinterpretations, and inaccurate conclusions.

In the next contribution, Pak et al. [27], starting from the fact that OS is an important mechanism underlying cellular damage of the inner ear resulting in hearing loss, reviewed the mechanisms underlying antioxidant-associated therapeutic effects. Noise exposure, ototoxic drugs, aging, and autoimmune damage seem to trigger inner ear injuries through increased ROS formation. Many compounds, such as n-acetylcysteine (combined with salicylate), alpha-lipoic acid, amifostine, ebselen, and coenzyme Q10, through different mechanisms, have displayed a preventive or rescuing impact against hearing loss in clinical studies. 

Manganosalen complexes are compounds that display elevated SOD, catalase, and peroxidase activities [28]. In their review, Rouco et al. [29] discuss the role of manganosalen in the prevention of neurodegenerative, inflammatory, and cardiovascular diseases, as well as liver, kidney, or lungs diseases. Despite the protective impact against ROS demonstrated by manganosalen complexes in OS models in vitro and in vivo, their potential use in humans continues to be questioned. 

Finally, Fratta Pasini et al. [30] reviewed the molecular pathogenesis of severe acute respiratory syndrome (SARS)-like coronavirus (SARS-CoV-2) and its relationship with OS and inflammation. They also discuss the potential adjuvant role of antioxidant and anti-inflammatory therapies to prevent SARS-CoV-2 complications. 

We would like to acknowledge the authors that contributed to this Special Issue. All studies emphasized the potential usefulness of antioxidant supplementation in different pathological situations, while also highlighting the need for further in vitro and in vivo studies. 
